# SPARC expression in patients with high-risk localized soft tissue sarcoma treated on a randomized phase II trial of neo/adjuvant chemotherapy

**DOI:** 10.1186/s12885-016-2694-2

**Published:** 2016-08-20

**Authors:** Elizabeth J. Davis, Lili Zhao, David R. Lucas, Scott M. Schuetze, Laurence H. Baker, Mark M. Zalupski, Dafydd Thomas, Rashmi Chugh

**Affiliations:** 1 Division of Hematology/Oncology, University of Michigan, 1500 E. Medical Center Dr., Ann Arbor, MI 48109 USA; 2 Department of Biostatistics, University of Michigan, 1500 E. Medical Center Dr., Ann Arbor, MI 48109 USA; 3 Department of Pathology, University of Michigan, 1500 E. Medical Center Dr., Ann Arbor, MI 48109 USA; 4 Department of Internal Medicine, Division of Hematology-Oncology, University of Michigan, 1500 E. Medical Center Dr., Ann Arbor, MI 48109 USA

**Keywords:** Soft tissue sarcoma, SPARC, Biomarker, Chemotherapy

## Abstract

**Background:**

Treatment for localized soft tissue sarcoma includes surgery and radiation, while the role of chemotherapy is controversial. Biomarkers that could predict therapeutic response or prognosticate overall survival (OS) are needed to define patients most likely to benefit from systemic treatment. Serum protein acidic and rich in cysteine (SPARC) is a matricellular glycoprotein that has been evaluated as a potential biomarker in numerous malignancies given its involvement in cell adhesion, proliferation, migration, and tissue remodeling.

**Methods:**

Using primary biopsy and resection specimens from patients with high-risk localized, soft tissue sarcoma treated on a neo/adjuvant chemotherapy study, SPARC expression was assessed and compared to patient and tumor characteristics, treatment, and outcomes. Survival functions were estimated using the Kaplan–Meier method and compared using the log-rank test. The Cox model was used for multivariate analysis.

**Results:**

Fifty patients had primary tumor specimens available. High, low, and no SPARC expression was found in 22, 13, and 15 patients, respectively. There was no significant difference in time to recurrence or OS between patients in these three groups. Comparing lack of SPARC expression with any SPARC expression, there was no significant difference in time to recurrence in patients without SPARC expression (*n* = 15) compared to patients with SPARC expression (*n* = 35). Likewise, there was no statistically significant difference in OS in patients without SPARC expression versus patients whose tumors expressed SPARC.

**Conclusions:**

Although we did not find a statistically significant difference in time to recurrence and OS in patients with high-risk soft tissue sarcoma, we did identify a trend toward improved time to recurrence and OS in patients whose tumors lacked SPARC expression. However, SPARC did not demonstrate the ability to discern which high-risk patients may have a worse prognosis or greater benefit from chemotherapy.

**Trial registration:**

The trial was registered on September 13, 2005 with ClinicalTrials.gov, number https://clinicaltrials.gov/ct2/show/NCT00189137?term=sarcoma&id=NCT00189137&state1=NA%3AUS%3AMI&phase=1&rank=1.

**Electronic supplementary material:**

The online version of this article (doi:10.1186/s12885-016-2694-2) contains supplementary material, which is available to authorized users.

## Background

Sarcomas are a heterogeneous group of malignant tumors of connective tissue and bone with a propensity for local recurrence and distant metastases. Surgery and radiation are the mainstays of treatment for localized disease while the role of chemotherapy in this setting is controversial. Novel biomarkers that could predict therapeutic response to chemotherapy or that could prognosticate overall survival (OS) are needed to define the patients most likely to benefit from systemic treatment.

Serum protein acidic and rich in cysteine (SPARC), also known as osteonectin and BM-40, is a matricellular glycoprotein that is secreted by tumor and/or surrounding stroma. SPARC has been evaluated as a potential biomarker in numerous malignancies given its involvement in cell adhesion, proliferation, migration, and tissue remodeling [1]. Studies in different cancer types have demonstrated varying effects on tumorigenesis. For example, in ovarian and colorectal cancer as well as neuroblastoma, SPARC has demonstrated antitumorigenic properties inhibiting angiogenesis and functioning as a tumor suppressor. Conversely, in breast, melanoma, brain, colon, prostate, kidney, esophageal, lung, and pancreatic cancers, SPARC expression has been associated with a more aggressive phenotype, inhibiting apoptosis and promoting tumor invasiveness and metastases [2]. The reasons for these disparate results, even within the same type of cancer, are unknown but may be explained by different methodologies used to assess SPARC expression as well as its complex biology. Studies that have evaluated different types of specimens including cell lines, xenografts, and patient samples could account for some variability. Discrepancies may also be due to the reporting of expression of tumoral versus stromal SPARC as the protein may have differential effects depending on the site of expression. Also, the peptides of SPARC that are present and interacting with the tumor microenvironment could account for the varying effects between cancer types [3].

The prognostic and predictive potential of SPARC in cancer is controversial. In one study of advanced pancreatic cancer patients receiving gemcitabine and nab-paclitaxel, high stromal SPARC expressers had a statistically significant longer OS than low SPARC expressers on multivariate analysis [4]. However, this finding was not confirmed on a subsequent phase III study [5]. In resected pancreatic cancer, SPARC stromal and cytoplasmic overexpression was associated with statistically significant worse disease-free survival (DFS) and OS in those treated with adjuvant gemcitabine, and not 5-fluorouracil, suggesting that SPARC may have a role as a predictive marker [6].

In breast cancer, SPARC expression has largely been associated with a more aggressive phenotype and an unfavorable prognosis [7, 8]. In a large study of localized breast cancer, pre-treatment core biopsies were analyzed for SPARC expression by IHC. The highest expression rates were seen in triple-negative tumors (TNBC). In the overall population as well as in the TNBCs, high SPARC expression was associated with a greater likelihood of pathological complete response to neoadjuvant chemotherapy with docetaxel, doxorubicin, and cyclophosphamide +/- capecitabine and vinorelbine suggesting a role as a predictive biomarker. There was no significant correlation between SPARC expression and DFS or OS [9].

In sarcoma, SPARC has recently been suggested as a potential prognostic factor in a relatively small study. Twenty-seven sarcoma specimens were obtained at varying time points in the patients’ clinical courses and evaluated for SPARC expression. Staining intensity ranged from 1+ (low) to 3+ (strong). Patients were identified as high SPARC expressers if at least 50 % of the tumor cells displayed 2+ staining (56 %). The other patients were considered low SPARC expressers (44 %). SPARC levels did not correlate with specific histologies but did correlate with OS. Low SPARC expressers had a median survival of 22.1 months while in the high expressers, the median survival was 4.4 months [10].

In this report, we sought to evaluate SPARC expression in a more uniform population of patients with high risk soft tissue sarcoma. Specifically, we performed an exploratory analysis of SPARC expression in patients with available tumor specimens that were enrolled on a randomized phase II study examining patient outcomes from treatment with one of two neo/adjuvant chemotherapy regimens for soft tissue sarcoma, doxorubicin and ifosfamide (AI) and gemcitabine and docetaxel (GT). Using primary biopsy and resection specimens, SPARC expression was evaluated in terms of patient and tumor characteristics, treatment, and outcome including OS and time to recurrent disease.

## Methods

### Study design

The associated phase II study was a randomized, open-label clinical trial in adults with stage IIB or III (localized, deep, >5 cm, high-grade) soft tissue sarcoma undergoing neo/adjuvant chemotherapy and evaluated the difference in the hospitalization rate between patients treated with the two regimens. Patients were randomized 1:1 to AI or GT. From November 2004 until August 2012, eighty patients were treated. Full study outcomes are reported elsewhere [11]. The trial is registered with ClinicalTrials.gov, number NCT00189137.

Archival tumor samples obtained as part of clinical care, through core needle biopsy to establish diagnosis or recurrence or at definitive surgery, were available for 57 patients. All samples were formalin-fixed paraffin-embedded. The samples were stored at the University of Michigan. All patients provided written informed consent to participate in the trial as well as for correlative work done on tumor and serum samples that were collected. The Institutional Review Board at the University of Michigan approved this study.

### Imunohistochemical staining of SPARC

Immunohistochemical staining (IHC) for SPARC was performed on formalin fixed, paraffin sections cut at 5 microns and rehydrated with water. Heat induced epitope retrieval was performed with FLEX TRS Low pH Retrieval buffer (6.10) for 20 min (Dako, N.A., Carpinteria, CA). After peroxidase blocking, the antibody SPARC (1B2) mouse monoclonal (Novus Biologicals, Littleton, CO) was applied at a dilution of 1:8000 at room temperature for 60 min. The FLEX HRP EnVision System (Dako N. A.) was used for detection. DAB chromagen was then applied for 10 min. Slides were counterstained with Harris Hematoxylin for 5 s, dehydrated, and coverslipped.

Immunostaining results were evaluated and scored by an expert sarcoma pathologist without knowledge of the clinical outcomes of the patients. IHC cutoffs for SPARC have not been established in sarcoma. Previous evaluation of SPARC expression in sarcoma has defined overexpression as a staining intensity of 2+ and percentage of cells stained ranging from 30 to 50 % [10, 12]. IHC results in our study were also assessed based upon staining intensity and percentage of cells stained. Staining intensity was scored in four levels, 0–3: 0 = none, 1 = weak, 2 = moderate, and 3 = strong. The percentage of cells that stained was scored 0–3: 0 = 0 %, 1 = 1–10 %, 2 = 11–50 %, 3 > 50 %. The individual scores were multiplied resulting in an immunoreactivity score ranging from 0 to 9. The cutoff for expression low versus high was chosen a priori to avoid potential bias. A score ≥ 4 was considered high and < 4 was low.

### Statistical analysis

Time to recurrence was defined as time from surgical resection to local or distant disease relapse. OS was defined as time from study entry to death of any cause. Survival functions were estimated using the Kaplan–Meier method and compared using the log-rank test. The Cox model was used for multivariate analysis. Clinicopathological variables including age, sex, tumor size, tumor site, histology, and chemotherapy regimen were included in the multivariate model. *P* values <0.05 from a two-sided test were considered as statistically significant.

## Results

There were 50 samples available from the primary sarcoma diagnosis and 17 samples available from disease recurrence. Ten patients had tumors from multiple time points available for analysis. Patient and sarcoma characteristics from the primary and recurrent tumors are detailed in Table 1. The most common histology evaluated in our study was undifferentiated pleomorphic sarcoma, and the most common tumor location was the extremity. Both primary and recurrent tumor samples were evaluated; however, there were fewer recurrent samples available for review limiting our statistical analysis of these specimens. Of the primary tumors examined, 49 were primary resection specimens, and one was a core needle biopsy. Seven of these specimens were obtained prior to chemotherapy, and the remaining 43 specimens were obtained after chemotherapy. Two specimens were also evaluated after radiation therapy. In the recurrent specimens, 13 specimens were obtained prior to treatment for recurrent disease. Four were obtained after chemotherapy had been administered.

**Table 1:** Patient and tumor characteristics

	Primary tumor (*n* = 50)	Recurrent tumor (*n* = 17)
Sex		
Male	34	12
Female	16	5
Median age in years (range)	61 (19–76)	50 (19–66)
Site of primary disease		
Extremity	30	10
Trunk	19	7
Head & Neck	1	0
Site of recurrent disease	NA	
Local		8
Distant		9
Histology		
Undifferentiated pleomorphic sarcoma (UPS)	32	6
Leiomyosarcoma (LMS)	6	1
Liposarcoma (LPS)	6	1
Synovial sarcoma (SS)	3	4
Malignant peripheral nerve sheath tumor (MPNST)	2	3
Fibrosarcoma (FS)	1	2
Prior treatment		
Gemcitabine/docetaxel	27	2
Doxorubicin/ifosfamide	17	1
Doxorubicin/dacarbazine	0	1
None	7	13
Radiation	2	0

**Fig. 1:**
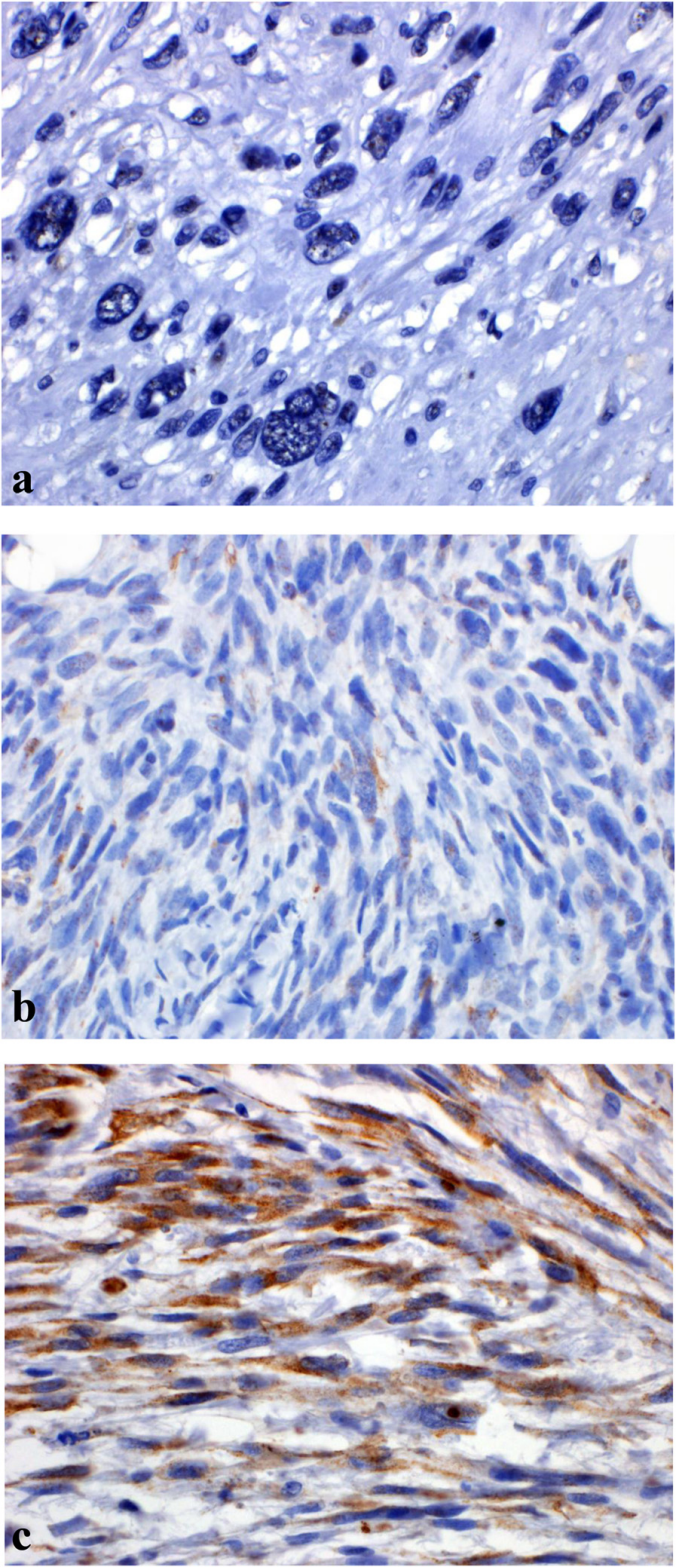
SPARC expression in 3 post-chemotherapy, primary resection patients

High, low, and no SPARC expression was found in 22, 13, and 15 primary sarcomas, respectively (see Table 2) and in 5, 4, and 8 recurrent sarcomas, respectively. Representative images from the primary resection of patients with leiomyosarcoma (no SPARC expression), malignant peripheral nerve sheath tumor (low SPARC expression), and myxofibrosarcoma (high SPARC expression) are shown in Fig. 1. All patients had undergone chemotherapy prior to their resection. HGUPS was the most common histology treated in our study (*n* = 32), and amongst those patients, SPARC expression level was distributed throughout the groups with 50, 28, and 22 % of tumors expressing high, low, and no SPARC. Although limited numbers in other histologies, SPARC expression was variable and showed no correlation with subtype, with the exception of the three synovial sarcoma specimens that all demonstrated a lack of SPARC expression.

**Table 2:** SPARC expression in the primary tumor

	High SPARC	Low SPARC	No SPARC
Histology	*N* = 22	*N* = 13	*N* = 15
UPS	16	9	7
LMS	2	2	2
LPS	3	1	2
Dedifferentiated	2	1	0
Pleomorphic/NOS	1	0	1
Myxoid	0	0	1
SS	0	0	3
MPNST	0	1	1
FS	1	0	0
Treatment for primary prior to tumor collection			
Gemcitabine/docetaxel	14	3	3
Doxorubicin/ifosfamide	5	9	10
None	3	1	3
Radiation	0	1	1
% necrosis observed in tumor after chemotherapy			
UPS	20–100	0–70	0–75
LMS	50	80^a^	30
LPS			
Dedifferentiated	15–50	0	NA
Pleomorphic/NOS	85	NA	10
Myxoid	NA	NA	90
SS	NA	NA	0–80^a^
MPNST	NA	5	40
FS	10	NA	NA
Best Response to chemotherapy by RECIST 1.0			
Partial response	0	2	0
Stable disease	19	8	14
Progressive disease	3	3	1
Recurrence	9	7	5
Local	2	4	0
Distant	7	3	5

^a^Pt with 80 % necrosis had prior radiation

In the primary sarcoma specimens, more patients with high SPARC expression had been pre-treated with GT, while more patients with no or low SPARC expression had been pretreated with AI. The best response to chemotherapy is listed in Table 2. Most patients had stable disease (SD) as their best response. There was no significant difference in SPARC expression based upon best response to chemotherapy as evaluated by Response Evaluation Criteria in Solid Tumors version 1.0 (RECIST) (data not shown).

**Fig. 2:**
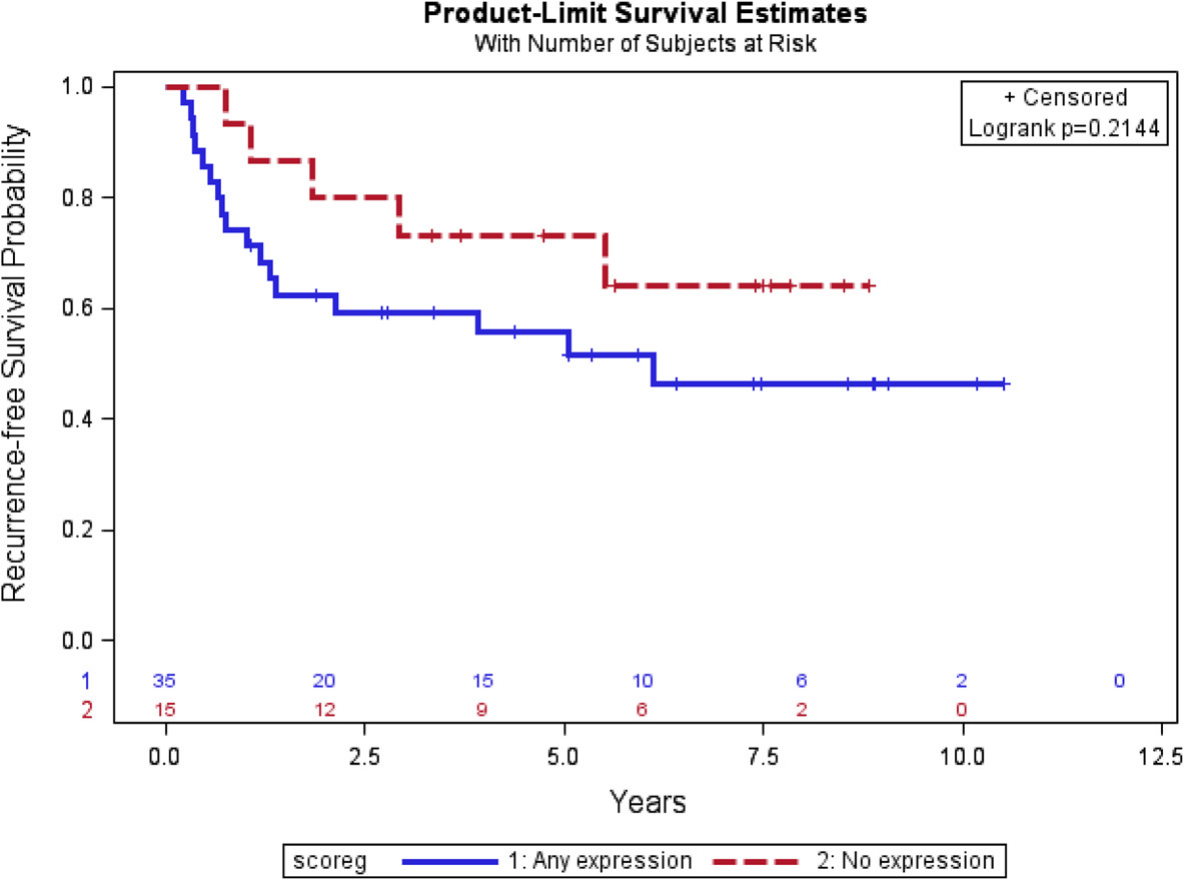
Recurrence-free survival by SPARC expression in primary tumors, none versus any SPARC

**Fig. 3:**
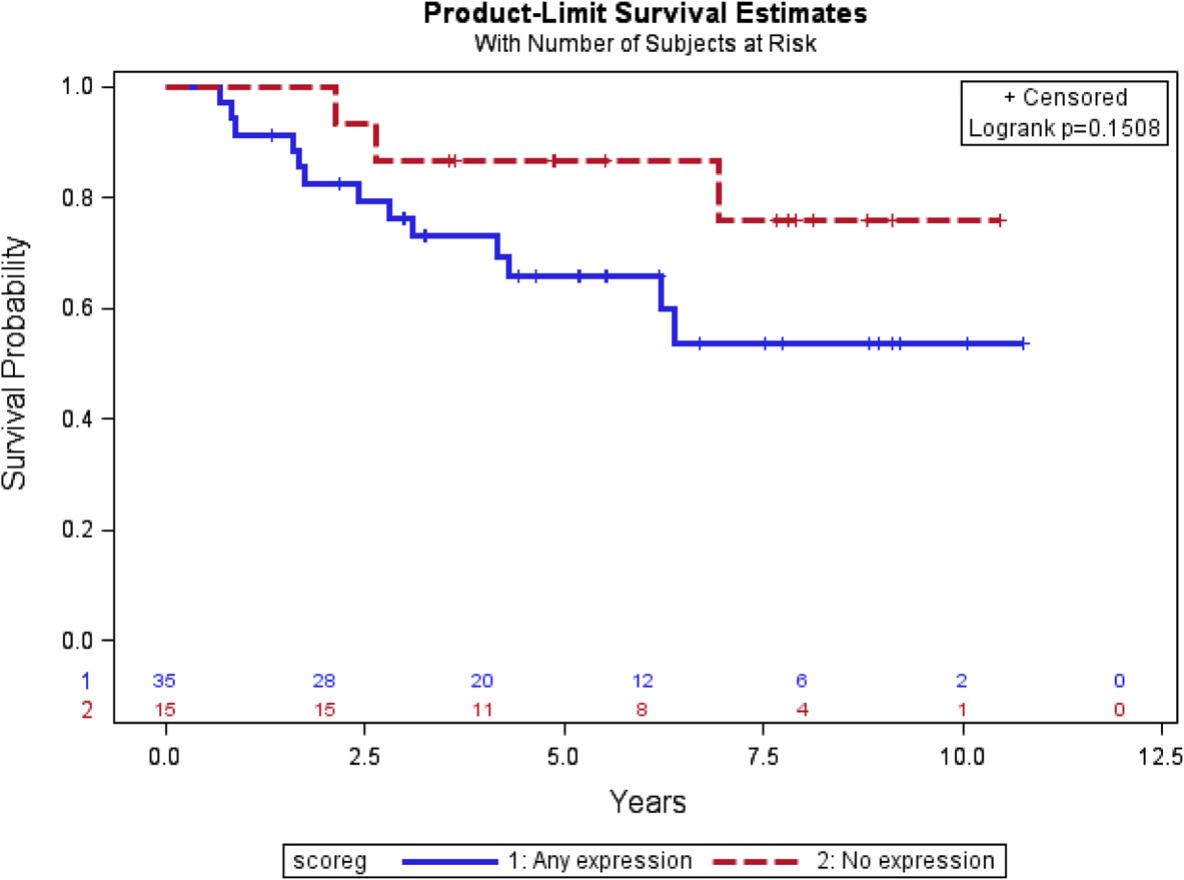
Overall survival by SPARC expression in primary tumors, none versus any SPARC

In order to analyze time to recurrent disease and OS, we divided the patients into two groups: those who expressed SPARC and those who did not express SPARC. In the primary tumor specimens, there was no statistically significant difference in time to recurrent disease in patients without SPARC expression (*n* = 15) than in those patients whose tumors expressed SPARC (*n* = 35), see Fig. 2. The 5 year relapse-free survival rates were 73 % (0.44–0.89) and 56 % (0.37–0.71) in the absence and presence of SPARC expression, respectively. Similarly, there was no difference seen in terms of the OS in sarcoma patients without SPARC expression compared to sarcoma patients with SPARC expression, see Fig. 3. With a median follow-up of 5 years, the median OS has not been reached in either group. The 5 year OS rates were 87 % (0.56–0.94, 95%CI) and 66 % (0.47–0.80) in the absence and presence of SPARC expression, respectively. The effect of SPARC expression on time to recurrence and OS was also not statistically significant when evaluated using a multivariate analysis including age, sex, tumor site, tumor histology, and chemotherapy regimen (see Additional file 1: Tables S1 & S2).

Differences in SPARC expression over time were also assessed for patients who had multiple tumor specimens available. There were ten patients included in this analysis. Six patients had no change in SPARC expression category (none, low, or high), although three of these patients did have a change in score within the same category. Three patients had a decrease in the expression category while one patient demonstrated an increased expression category. These changes were not statistically significant. See Table 3 for complete characterization of these results.

**Table 3:** Changes in SPARC expression over time

Histology	Primary site	Primary SPARC score	Treatment prior to primary score	Local (L) or distant (D) recurrence	Secondary SPARC score	Treatment prior to secondary score
MPNST	Extremity	0	AI	D	0	AD
SS	Extremity	0	GT, XRT	D	0	None
MPNST	Extremity	1	AI	L	0	None
UPS	Extremity	1	AI	D	2	GT
LMS	Trunk	1	AI, XRT	D	0	GT
UPS	Trunk	2	GT	L	2	None
UPS	Trunk	2	AI	L	4	None
Fibrosarcoma	Extremity	4	AI	L	6	None
Dediff LPS	Trunk	6	GT	D	4	None
UPS	Extremity	9	GT	D	0	None

A- doxorubicin, I- ifosfamide, G-gemcitabine, T- docetaxel, D- dacarbazine

## Discussion

In this study, we analyzed SPARC expression in sarcoma patients and found that 70 % of tumors demonstrated some SPARC expression, including 44 % with high SPARC expression. There was no statistically significant difference seen in time to recurrence and OS in patients whose tumor lacked SPARC expression versus those who expressed SPARC.

While SPARC expression did not show a statistical significant correlation with overall survival or disease free survival, there was some difference noted. Our results did show a trend similar to the Morgan et al. sarcoma study which found that high SPARC expression was associated with a shorter OS than low SPARC expression [10]. However, the magnitude of difference was not nearly as robust as reported previously, even in the absence of significance, and whether additional patients or follow up time would have an impact on this potential trend is unknown. All of our patients had high-risk disease (stage IIB or III) as defined by widely accepted clinical parameters including tumor size and grade. This subset of sarcoma patients is at the highest risk of metastases and thus is often considered for adjuvant chemotherapy. Given the rarity, heterogeneity, and lack of large, randomized studies in the disease, the role of adjuvant chemotherapy in soft tissue sarcoma remains controversial. Finding a prognostic biomarker or a predictive factor of benefit for adjuvant therapy in this group would be of great value in assisting clinicians in making these difficult recommendations. While SPARC expression did suggest the ability to prognosticate in a small, heterogeneous study, in our study including this more uniform high-risk population, in which the use of a biomarker would arguably be most useful, SPARC did not prove to be informative.

Compared with other malignancies, there is limited data on SPARC expression in sarcoma, and there is no uniformly accepted determinant of high vs. low expression. A recent large study of a varied group of 2539 bone and soft tissue sarcoma specimens evaluated SPARC expression by IHC using a cutoff of ≥ 2+ and ≥ 30 % for positivity. SPARC overexpression was seen in 35.9 % of the cases, with the highest percentages in epithelioid hemangioendothelioma, conventional chondrosarcoma, and angiosarcoma. In this study, no clinical information in terms of treatment, extent of disease, or survival was reported [12]. This report raised the possibility of increased SPARC expression in vascular sarcomas as compared to other sarcoma subtypes but did not comment on its prognostic or predictive value.

Another study, focused solely on osteosarcoma patients, evaluated SPARC by quantitative real-time PCR in initial diagnostic biopsies, primary resections, and metastatic disease. High SPARC expression was found in the majority of specimens, 51/55 (96.3 %) and correlated with a poorer event free-survival (*p* = 0.03) and relapse free survival (*p* = 0.07). There was no difference in OS [13]. These findings suggest a potential role for SPARC as a prognostic biomarker in osteosarcoma, although with the majority of patients expressing SPARC, the clinical implications are unclear. Similar to our results in soft tissue sarcoma, SPARC overexpression in osteosarcoma did not correlate with different time points of specimen collection (biopsy, surgical resection, and metastasis) or clinical and pathological variables such as age, gender, histology, or site of primary disease.

Because SPARC mediates albumin transport, it may facilitate entry of albumin-bound paclitaxel into the tumor cells [3]. This has led to studies in various malignancies evaluating the efficacy of nanoparticle albumin-bound (nab)-paclitaxel based upon SPARC expression with correlation in response rates in high SPARC expressers in head and neck cancer patients [14] but inconsistent results in pancreatic cancer patients [5]. The evaluation of efficacy of nab-paclitaxel in sarcoma has thus far been limited to one small phase II study of 15 assessable patients [15]. Various subtypes were included and SPARC expression was not assessed, and given the small sample size and heterogeneity of tumor types, no conclusions as to the efficacy of nab-paclitaxel in sarcoma could be drawn from this study. Preclinical work in xenograft models of osteosarcoma and Ewing’s sarcoma has demonstrated inhibition of tumor growth by nab-paclitaxel [16]. As nab-paclitaxel or other chemotherapy agents that bind to albumin such as aldoxorubicin are evaluated in sarcoma, the use of SPARC as a predictive biomarker might be considered.

There were limitations to our study. The small sample size precludes adequate power to detect differences in SPARC expression between the various subtypes of sarcoma. There also were no vascular or bone sarcomas in our dataset which may be subtypes for which SPARC has higher prognostic potential. Our data are also limited in that nearly all samples were post-chemotherapy, and it is entirely possible that chemotherapy changes SPARC expression levels [17]. It is also conceivable that the treatment rendered on clinical study influenced patient outcomes independent of SPARC expression. In an optimal setting, SPARC expression would be determined pre and post-treatment in order to discern its prognostic and predictive significance. The optimal SPARC antibody also needs to be determined as the studies performed thus far have used different antibodies. Standardized scoring of SPARC expression and defining what constitutes overexpression is also important for the design of subsequent work. Future studies might focus on prospective evaluation of SPARC expression in sarcoma, especially in a cohort of patients treated with albumin-bound agents.

## Conclusions

In conclusion, while this study found a trend toward improved time to recurrence and OS in patients with high-grade soft tissue sarcoma whose tumors lacked SPARC expression, SPARC did not demonstrate the ability to discern which high-risk patients may have a worse prognosis or a greater benefit from chemotherapy.

## Additional file

Additional file 1:
**Table S1.** Multivariate model for OS by SPARC expression, any versus no expression. **Table S2.** Multivariate model for time to recurrence by SPARC expression, any versus no expression. **Table S3.** Distribution of SPARC scores. (DOCX 15 kb)

## Abbreviations

AI: Doxorubicin/ifosfamide
DFS: Disease-free survival
FS: Fibrosarcoma
GT: Gemcitabine/docetaxel
IHC: Immunohistochemical staining
LMS: Leiomyosarcoma
LPS: Liposarcoma
MPNST: Malignant peripheral nerve sheath tumor
Nab: Nanoparticle albumin-bound
OS: Overall survival
RECIST: Response evaluation criteria in solid tumors
SD: Stable disease
SPARC: Serum protein acidic and rich in cysteine
SS: Synovial sarcoma
UPS: Undifferentiated pleomorphic sarcoma
