# Supplementary material for: SPARC expression in patients with high-risk localized soft tissue sarcoma treated on a randomized phase II trial of neo/adjuvant chemotherapy
Source: BMC Cancer. 2016 Aug 20;16:663. doi: 10.1186/s12885-016-2694-2 (PMC4992190; doi:10.1186/s12885-016-2694-2)
Supplement: Additional file 1: — Table S1. Multivariate model for OS by SPARC expression, any versus no expression. Table S2. Multivariate model for time to recurrence by SPARC expression, any versus no expression. Table S3. Distribution of SPARC scores. (DOCX 15 kb) [file 12885_2016_2694_MOESM1_ESM.docx]

| **Variable** |  | **HR** | **95% CI limits** | | **p value** |
| --- | --- | --- | --- | --- | --- |
| **score** | **Any vs No expression** | 2.599 | 0.511 | 13.209 | 0.2495 |
| **Age** |  | 1.031 | 0.975 | 1.089 | 0.2828 |
| **Size** |  | 1.051 | 0.93 | 1.189 | 0.424 |
| **Sex** | **F vs M** | 2.417 | 0.661 | 8.831 | 0.182 |
| **Treatment** | **AI vs non-AI** | 2.277 | 0.685 | 7.564 | 0.1792 |
| **Primary Site** | **Head vs. non-Head** | 3.21E-15 | 0 | Infinity | 0.9943 |
|  | **extremity vs. non-extremity** | 0.1823 | 0.0189 | 1.7598 | 0.1412 |
|  | **trunk vs. non-trunk** | 1.71E+15 | 0 | Infinity | 0.994 |
| **Histology** | **Fibrosarcoma vs. non-Fibrosarcoma** | 1.33E-13 | 0 | Infinity | 0.9949 |
|  | **LMS vs. non-LMS** | 0.1013 | 0.0013 | 7.9207 | 0.3033 |
|  | **LPS vs. non-LPS** | 0.7949 | 0.0072 | 87.8212 | 0.9238 |
|  | **MPNST vs. non-MPNST** | 85.7261 | 0.1003 | 73272.5 | 0.1962 |
|  | **SS vs.non-SS** | 209.8 | 0.8258 | 53307.8 | 0.0585 |
|  | **UPS vs.non-UPS** | 5.19E+09 | 0 | Infinity | 0.9962 |

Table S1. Multivariate model for OS by SPARC expression, any versus no expression

Table S2. Multivariate model for time to recurrence by SPARC expression, any versus no expression

| **Variable** |  | **HR** | **95% CI limits** | | **p value** |
| --- | --- | --- | --- | --- | --- |
| **score** | **Any vs No expression** | 2.279 | 0.599 | 8.668 | 0.2267 |
| **Age** |  | 1.039 | 0.987 | 1.094 | 0.1432 |
| **Size** |  | 1.064 | 0.945 | 1.199 | 0.3066 |
| **Sex** | **F vs M** | 3.315 | 1.014 | 10.835 | 0.0474 |
| **Treatment** | **AI vs non-AI** | 2.897 | 0.936 | 8.963 | 0.0649 |
| **Primary Site** | **Head vs. non-Head** | 0.2287 | 0.00035 | 149.6 | 0.6556 |
|  | **extremity vs. non-extremity** | 0.3684 | 0.0421 | 3.2209 | 0.3667 |
|  | **trunk vs. non-trunk** | 11.8695 | 0.00628 | 22424 | 0.5204 |
| **Histology** | **Fibrosarcoma vs. non-Fibrosarcoma** | 5.7127 | 0.0555 | 588.1 | 0.4611 |
|  | **LMS vs. non-LMS** | 0.4443 | 0.0142 | 13.873 | 0.644 |
|  | **LPS vs. non-LPS** | 1.1663 | 0.00988 | 137.7 | 0.9496 |
|  | **MPNST vs. non-MPNST** | 5649.8 | 12.0633 | 3E+06 | 0.0059 |
|  | **SS vs.non-SS** | 87.2368 | 0.5123 | 14856 | 0.0882 |
|  | **UPS vs.non-UPS** | 6.85E-07 | ####### | 0.2506 | 0.0299 |

Table S3. Distribution of SPARC scores

| Score | Number of patients |
| --- | --- |
| 0 | 15 |
| 1 | 6 |
| 2 | 6 |
| 3 | 1 |
| 4 | 8 |
| 6 | 7 |
| 9 | 7 |
